# Donor Dilemma: A National Simulation‐Based Case Curriculum for Teaching Donor Call Decision‐Making in Infectious Diseases Trainees

**DOI:** 10.1111/tid.70284

**Published:** 2026-07-21

**Authors:** Robert Sean Tyler, Rachel B. Colven, Alexandra G. Mills, Drew W. Charles, Ruth O. Adekunle, Divisha Sharma, Yosra Alkabab, Scott R. Curry, Ricardo M. La Hoz, Courtney E. Harris

**Affiliations:** ^1^ Division of Infectious Diseases Medical University of South Carolina Charleston South Carolina USA; ^2^ Department of Pharmacy Medical University of South Carolina Charleston South Carolina USA; ^3^ Division of Infectious Diseases UT Southwestern Medical Center Dallas Texas USA

**Keywords:** donor call, medical education, simulation, transplant infectious diseases

## Abstract

**Introduction:**

Donor‐call decision‐making during organ allocation requires clinical reasoning in the setting of uncertainty, time pressure, and evolving information. In transplant infectious diseases (TID), this skill is essential, yet exposure during training is often inconsistent and informal. Educational strategies that allow trainees to practice this type of decision‐making remain limited. We aimed to evaluate the feasibility, acceptability, and perceived impact of an asynchronous electronic, case‐based progressive disclosure curriculum designed to support clinical reasoning during donor‐call decision‐making among infectious diseases (ID) trainees.

**Methods:**

In this pilot feasibility study, we developed a self‐paced, asynchronous donor‐call curriculum consisting of 12 case‐based modules. Cases reflected real‐world donor scenarios. After national dissemination to ID and TID training programs, participants completed all modules and a post‐curriculum survey assessing feasibility and perceived educational impact.

**Results:**

Around 29 trainees from 22 academic medical centers completed all 12 modules. Most had little prior donor‐call exposure. Eighty‐six percent endorsed greater confidence in their clinical reasoning, and 97% reported greater confidence in assessing donor suitability. The curriculum was feasible within clinical workflows, and 97% of participants indicated they would recommend it to their peers. Perceived gains were most pronounced among trainees with minimal transplant experience, with benefit remaining high even among those in dedicated TID training years.

**Conclusion:**

An asynchronous donor‐call case‐based progressive disclosure curriculum was feasible and valued across diverse ID and TID training programs. This scalable approach may offer a framework for supporting clinical reasoning in other areas of medicine where complex decisions must be made quickly with limited information.

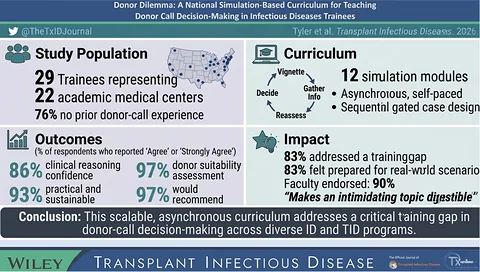

## Graphical abstract

An asynchronous donor‐call case‐based progressive disclosure curriculum was feasible and valued across diverse ID and TID training programs. ID and TID trainees reported improved confidence in their clinical reasoning and ability to evaluate donor‐suitability, supporting this educational approach to address a training gap.

**Abbreviations**
ID: infectious diseases
TID: transplant infectious diseases

## 1 Introduction

Physicians must routinely integrate incomplete and evolving information, navigate competing risks, and arrive at judgments in situations where no single answer exists. Donor‐derived infectious risk assessment during organ allocation exemplifies this challenge. It requires rapid synthesis of limited data while balancing the risk of transmissible infection against organ non‐utilization and prolonged waitlist morbidity and mortality. Deceased donor evaluation is a core responsibility within transplant infectious diseases (TID). Donor‐derived infections, while uncommon, can result in severe recipient outcomes, with graft loss or death occurring in approximately one‐third of affected patients [1]. These consultations, referred to as “donor calls,” occur under significant time pressure, outside of routine clinical workflows, and with limited access to complete donor histories, creating substantial cognitive burden for the evaluating physician [2].

Despite the importance of donor evaluation as a core competency in TID, exposure to donor calls during training is inconsistent and subject to the “hidden curriculum” where exposure depends heavily on institutional volume and informal participation rather than standardized training. Many infectious diseases (ID) and TID fellows complete training with limited or no direct experience participating in donor evaluations. Even when exposure occurs, teaching is often informal and episodic, with limited opportunity for deliberate practice or structured feedback, and prior educational work has emphasized the need for principled frameworks to guide donor‐call decision‐making [2].

Simulation‐based education has been increasingly used to address this training gap. Sigler et al. developed a donor call simulation delivered via real‐time paging or text messaging during clinical rotations, demonstrating that trainees perceived the activity as effective [3]. Notably, this work also revealed discordance between seemingly correct accept‐or‐decline decisions and the underlying clinical reasoning in a substantial proportion of cases. While this simulation provided valuable insights into trainee reasoning, its implementation required synchronous faculty participation and occurred within the constraints of active clinical service.

To address this gap, we developed a self‐paced, online, asynchronous donor call progressive disclosure curriculum intended to operationalize clinical reasoning in uncertain clinical contexts. We conducted a national pilot to assess feasibility, acceptability, and perceived educational impact among ID fellows, including those pursuing additional training in TID.

## 2 Methods

### 2.1 Curriculum Development and Structure

We developed a series of 12 case‐based donor call modules for ID and TID trainees with a total estimated completion time of approximately 1 h based on initial pilot testing. The curriculum was created in‐house at the Medical University of South Carolina using the Articulate Rise 360 platform, an asynchronous online learning platform. Cases were drawn from prior clinical encounters and selected and mapped to a curriculum blueprint designed to capture common and high‐stakes donor‐derived infectious risk categories and core clinical reasoning domains relevant to donor evaluation (Table 1). All cases and accompanying content were reviewed by seven TID faculty physicians, including one from an outside institution, and a TID clinical pharmacist. Case content and learning objectives were defined by multidisciplinary faculty consensus and aligned with contemporary practice standards.

**TABLE 1:** Curriculum blueprint of cases mapped to high‐risk infectious scenarios.

Case	Donor scenario	Infectious risk category	Core teaching focus	Cognitive skill
(1)	Donor with recently treated cryptococcal meningitis and pneumonia receiving antifungal therapy at the time of donation	Donor‐derived invasive fungal infection (*Cryptococcus*)	Assess transplant suitability and determine recipient antifungal management when donors have recent or partially treated cryptococcosis	Infectious risk stratification
(2)	Donor with persistent MRSA bacteremia despite therapy (PWID; repeat cultures positive; TTE without vegetations)	Donor‐derived bacterial bloodstream infection with concern for infective endocarditis (MRSA)	Assess transplant suitability when persistent bacteremia raises concern for endocarditis or metastatic infection and determine recipient antibiotic management	Infectious risk stratification
(3)	Donor with multiple intracranial lesions on imaging and epidemiologic risk factors for endemic infection	Potential donor‐derived parasitic or endemic fungal infection (e.g., neurocysticercosis, coccidioidomycosis)	Evaluate CNS lesions in donors and determine when targeted infectious screening or recipient prophylaxis is warranted	Epidemiologic risk assessment
(4)	Donor with unexplained meningoencephalitis (altered mental status, CSF pleocytosis) and seasonal or exposure risk factors	High‐risk donor‐derived CNS infection (encephalitis of unclear etiology)	Determine when donors with unexplained encephalitis should be declined and distinguish contraindicated CNS infections from treatable bacterial meningitis scenarios	Diagnostic interpretation and decision‐making under uncertainty
(5)	Donor with ESBL‐*Klebsiella pneumoniae* bacteremia following septic shock prior to donation	Donor‐derived bacterial bloodstream infection	Assess transplant suitability and determine appropriate recipient antimicrobial management when donors have treated bacteremia	Infectious risk stratification
(6)	Donor with a positive RPR identified during routine infectious screening	Potential donor‐derived bacterial infection (*Treponema pallidum*)	Confirm nontreponemal screening with treponemal testing and determine recipient management when treatment history is uncertain	Evidence interpretation
(7)	Donor with an incidentally discovered cavitary pulmonary nodule on imaging	Potential donor‐derived pulmonary infection (e.g., mycobacterial or fungal infection)	Evaluate unexplained pulmonary lesions in donors and determine when organ donation should be deferred pending further microbiologic evaluation	Diagnostic interpretation and decision‐making under uncertainty
(8)	Transplant candidate with a positive SARS‐CoV‐2 PCR prior to organ acceptance	Active viral infection in transplant candidate (SARS‐CoV‐2)	Determine whether transplantation should proceed in the setting of active COVID‐19 and interpret Ct values within the broader clinical context	Evidence interpretation and risk stratification
(9)	Donor with a dermatomal vesicular rash consistent with varicella zoster virus (VZV)	Donor‐derived viral infection risk (VZV)	Assess transplant suitability and determine appropriate recipient prophylaxis when donors have active herpes zoster	Infectious risk stratification
(10)	Donor with respiratory cultures growing mold after a prolonged ICU course	Donor‐derived filamentous fungal infection (e.g., *Aspergillus*, *Mucorales*)	Distinguish airway colonization from invasive disease and determine whether organ acceptance is appropriate	Diagnostic interpretation and risk stratification
(11)	Donor with confirmed HSV encephalitis (CSF HSV PCR positive with compatible MRI findings)	Donor‐derived viral infection risk (Herpes simplex virus)	Assess organ suitability and determine appropriate recipient antiviral management when donors have treated HSV encephalitis	Infectious risk stratification
(12)	Donor with a prior diagnosis of chikungunya identified during donor evaluation	Potential donor‐derived arboviral infection (e.g., chikungunya, dengue, Zika)	Assess transmission risk and determine whether deferral or additional testing is warranted when donors have a history of arboviral infection	Epidemiologic risk assessment

Abbreviations: CNS, central nervous system; COVID‐19, coronavirus Disease of 2019; CSF, cerebrospinal fluid; Ct, cycle threshold; ESBL, extended‐spectrum beta‐lactamase; ICU, intensive care unit; MRI, magnetic resonance imaging. MRSA, methicillin‐resistant *Staphylococcus aureus*; PCR, polymerase chain reaction; PWID, person who injects drugs; RPR, rapid plasma reagin; SARS‐CoV‐2, Severe Acute Respiratory Syndrome Coronavirus 2; TTE, trans‐thoracic echocardiography.

Each module began with a brief clinical vignette reflecting the uncertainty and limited information typical of donor‐call decision‐making. Following the vignette, learners engaged in a structured interactive multiple‐choice exercise focused on stepwise information gathering. Learners selected which additional data to request, including donor social and exposure history, laboratory results, imaging findings, and elements of the hospital or clinical course. Information was released sequentially within each case, requiring learners to reassess their decisions as additional information became available, ultimately culminating in a decision regarding organ acceptability (Figure 1) and case resolution. This also intentionally included cases where specific lab values or results were delayed or unavailable before donation.

**FIGURE 1:**
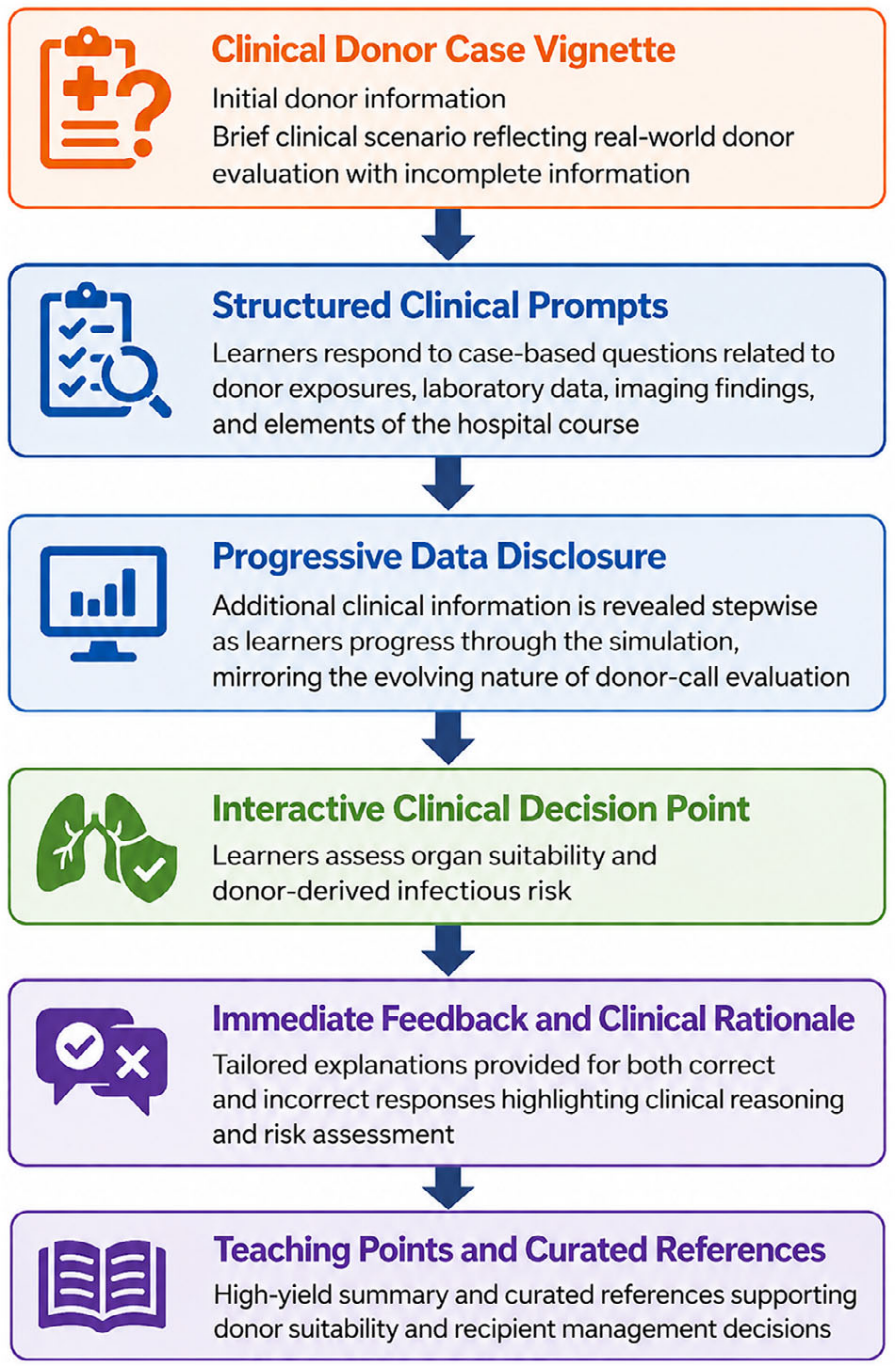
Learning schema.

The simulations used a sequential, gated format. Learners were required to select an acceptable response before advancing. In recognition of practice heterogeneity, more than one response could be acceptable depending on the clinical context. This approach was intended to expose learners to practice variability. When an incorrect response was selected, tailored feedback explained the rationale and prompted reconsideration before learners proceeded. Responses did not lead to divergent case pathways. Modules concluded with high‐yield teaching points and curated literature references and could be repeated for additional practice.

Before national dissemination, the curriculum was piloted in‐house at our institution. Pilot feedback informed minor refinements to the case flow, wording, and clarity. Pilot participants were included in the final analytic cohort.

### 2.2 Curriculum Design

A central design principle of the curriculum was the recognition of practice heterogeneity as an inherent feature of donor call decision‐making in TID. Approaches to donor acceptance vary across transplant centers based on institutional risk tolerance, organ availability, recipient populations, and local expertise. Rather than attempting to standardize these decisions, the curriculum was designed to expose trainees to this variability.

Situated learning served as the primary pedagogical framework, with principles drawn from script concordance theory informing case structure [4, 5]. The cases present ill‐defined clinical problems requiring learners to update judgments as new information becomes available.

### 2.3 Participant Recruitment and Implementation

Program directors of ID and TID training programs in the United States were contacted by email and were invited to share access to the donor call modules with trainees. We aimed to contact accredited US ID and TID training programs using publicly available program listings and professional networks. Academic and community programs were contacted with the aim of recruiting diverse geographic and institutional representation across various training environments. Recruitment emails were sent in mid‐July 2025 and again in January 2026, with module and survey access ending February 12, 2026.

Eligible participants included both general ID and TID fellows. ID and TID faculty who independently requested access to the modules were permitted to complete the same post‐curriculum survey to assess perceived educational value and feasibility.

Participation was voluntary, and sign‐up and access were free. Learners self‐enrolled using a direct link or QR code included in the recruitment email. The modules were available from June 15, 2025, through February 12, 2026. Sequential completion of all modules was required to access the post‐curriculum survey.

### 2.4 Survey and Outcomes Assessments

After completing the final module, learners were directed to a Research Electronic Data Capture (REDCap) survey. Responses were de‐identified and aggregated. Survey items were developed by TID faculty and mapped to the curriculum objectives, assessing perceived impact and feasibility consistent with evaluation at Kirkpatrick Level 1 (learner reaction) [6]. Demographic information including gender, postgraduate year of training, prior time spent on immunocompromised host rotations, and prior donor call exposure were collected. Educational impact was assessed using a five‐point Likert scale evaluating perceived benefit and usefulness of the curriculum and likelihood of recommending the modules to a colleague. All submitted survey responses were included in the analysis. Data were summarized descriptively with Likert responses reported as percentages.

Individual learner response selections from the simulation were not captured due to the gated design, and no scoring rubric was applied, consistent with the formative focus of the curriculum. This study was reviewed by the Institutional Review Board at our institution and determined to be exempt (Protocol #00141889). Informed consent was implied by voluntary participation.

## 3 Results

Around 29 trainees from 22 academic medical centers completed all 12 modules and the post‐curriculum survey (Tables 2 and 3). Respondents included trainees from academic medical centers across the United States, including South Carolina (*n* = 4), Maryland (*n* = 4), Texas (*n* = 3), North Carolina (*n* = 2), Missouri (*n* = 2), and individual respondents from California, New Hampshire, New Jersey, Alabama, Virginia, Michigan, Wisconsin, Tennessee, Washington, DC, Minnesota, Indiana, and Pennsylvania. All respondents who reported institutional affiliation (27 of 29 respondents) were from academic medical centers. Note that 19 of 29 respondents were female (66%). Most respondents were first‐ and second‐year ID fellows (9 and 11 trainees, 31% and 38%, respectively), followed by third‐year fellows (6 trainees, 21%) and fourth‐year fellows in extended or research training years (3 trainees, 10%). Eight trainees (28%) were in dedicated TID training years. The pooled median time spent on a transplant or immunocompromised host service was 8 weeks (IQR: 4–16). Among general ID fellows not in a dedicated TID training year, median service time was 6 weeks (IQR: 3.5–10.5), compared with 27 weeks (IQR: 6–39) among those in dedicated TID training years. Among respondents reporting career plans, 15 of 29 (52%) intended to pursue TID. Prior exposure to donor‐call education was limited, with 22 of 29 trainees (76%) reporting no prior donor‐call experience, and the remaining trainees reporting primarily informal, ad hoc discussions.

**TABLE 2:** Participant characteristics among trainees completing modules (*N* = 29).

Characteristic	*n* (%)
Sex	
Female	19 (66)
Male	10 (34)
Year of ID fellowship^a^	
Year 1	9 (31)
Year 2	11 (38)
Year 3	6 (21)
Year 4	3 (10)
Median time on transplant/immunocompromised host service	8 weeks (IQR: 4–16)
Plan to pursue transplant infectious diseases	
Yes	15 (52)
No	14 (48)
Dedicated TID training year	
Yes	8 (28)
No	21 (72)
Prior donor call exposure	
Yes	7 (24)
No	22 (76)

^a^ Fellowship year reflects current training year; dedicated transplant infectious diseases (TID) training is reported separately.

**TABLE 3:** Trainee perceptions of educational impact, feasibility, and engagement reported as agree/strongly agree responses.

Outcome measure	All trainees (*N* = 29), *n* (%)	General ID fellows not in a dedicated TID year (*N* = 21), *n* (%)	Dedicated TID Year (*N* = 8), *n* (%)
Educational impact			
Improved ability to assess donor suitability	28 (97)	21 (100)	7 (88)
Enhanced clinical reasoning skills	25 (86)	19 (90)	6 (75)
Curriculum addressed a gap in TID training	24 (83)	20 (95)	4 (50)
Preparedness for clinical practice			
Prepared to apply knowledge to donor call scenarios	24 (83)	18 (86)	6 (75)
Knowledge reinforcement			
Teaching points reinforced key concepts	27 (93)	21 (100)	6 (75)
Feasibility and sustainability			
Time required to complete modules appropriate	28 (97)	21 (100)	7 (88)
Curriculum practical and sustainable for training	26 (93)^a^	20 (95)	6 (86)^b^
Engagement and acceptability			
Would recommend modules to co‐fellows	28 (97)	21 (100)	7 (88)
Would revisit modules in the future	24 (83)	18 (86)	6 (75)

^a^ Among respondents, *n* = 28.

^b^ Among respondents, *n* = 7.

The curriculum addressed a perceived training gap for 24 of 29 trainees (83%), with 25 of 29 (86%) reporting greater confidence in their clinical reasoning and 28 of 29 (97%) reported improved understanding of donor suitability assessment. Feasibility outcomes were positive. Twenty‐eight of 29 trainees (97%) reporting that the time required to complete the modules was appropriate and 26 of 28 (93%) viewing the curriculum as practical and sustainable within clinical training workflows.

Preparedness for clinical practice was also assessed. Most trainees agreed or strongly agreed that they felt prepared to apply the knowledge gained to real‐world donor‐call scenarios, with 24 of 29 trainees (83%) reporting this. A total of 27 of 29 trainees (93%) reported improved understanding of post‐acceptance management considerations for transplant recipients. Engagement was strong, with 28 of 29 participants (97%) indicating they would recommend the curriculum to co‐fellows, and 24 of 29 trainees (83%) reporting they would revisit the modules in the future when encountering similar cases.

Among trainees in dedicated TID training years, six of eight trainees (75%) reported enhanced clinical reasoning and preparedness for donor‐call evaluations, while seven of eight trainees (88%) endorsed improved donor suitability assessment. Six of seven trainees (86%) endorsed curriculum feasibility (Table 3). A smaller proportion of trainees in dedicated TID years felt the curriculum addressed a training gap (50%), which may reflect a greater baseline exposure to donor‐call decision‐making.

Trainees frequently commented on the realism and practicality of the cases. Several trainees highlighted the modules’ ability to simulate time‐pressured donor‐call environments and translate complex clinical considerations into clearer decision frameworks. One trainee described the curriculum as making “an intimidating and huge topic digestible.”

While evaluation by faculty was not a primary objective of this study, 10 TID faculty representing seven academic medical centers independently requested access to the curriculum and completed the post‐curriculum survey. Also, 9 of 10 faculty (90%) reported enhanced clinical reasoning, improved preparedness for donor‐call decision‐making, and indicated they would recommend the curriculum to fellows and junior attending physicians. All faculty respondents reported that the curriculum was feasible and appropriate for incorporation into clinical training workflows. One faculty member noted, “We have been considering ways to improve donor call training, and this is a helpful resource.” Another noted that the curriculum would be helpful for fellows and junior attending physicians covering transplant services.

## 4 Discussion

This multicenter pilot feasibility study suggests that an asynchronous electronic, case‐based progressive disclosure curriculum is a feasible and well‐accepted approach to teaching clinical reasoning in high‐stakes, uncertain clinical contexts within TID training. Across national institutions, trainees and faculty alike reported that the curriculum addressed a meaningful gap in training and was associated with perceived improvements in clinical reasoning and preparedness for real‐world donor‐call decision‐making. For nearly 80% of trainees, this represented their first structured exposure to donor evaluation, highlighting the limitations of relying on informal clinical participation alone.

Prior educational efforts in donor call training have largely depended on simulation embedded within clinical workflows [2, 3]. While effective, these approaches require real‐time faculty involvement and are often limited by competing clinical demands. The asynchronous design of this curriculum was developed to overcome these barriers. Learners were able to engage in repeated, deliberate practice on their own schedules while still encountering the uncertainty, evolving information, and cognitive load that are inherent in real‐world donor evaluations. The sequential, gated format was designed to promote active information gathering and iterative risk assessment rather than passive case review.

An important feature of the curriculum was the deliberate inclusion of practice heterogeneity. Donor acceptance decisions vary widely across transplant centers based on institutional risk tolerance, organ availability, and patient populations. Rather than prescribing a single “correct” approach, the modules exposed trainees to multiple acceptable management pathways grounded in principled reasoning. This more closely reflects real clinical practice and may help prepare learners to navigate nuanced decision‐making across diverse training environments. This was also grounded in a structured curriculum blueprint that mapped cases to high‐risk donor‐derived infectious scenarios and core cognitive skills relevant to donor evaluation (Table 1). This helped ensure that the cases covered a range of clinically meaningful situations while reinforcing key domains of clinical reasoning, rather than relying on more ad hoc case selection.

Feasibility and acceptability outcomes were also consistently strong. The curriculum required no synchronous faculty facilitation during implementation and leveraged an existing online learning platform, allowing broad participation with minimal ongoing resource burden. High engagement and strong willingness to recommend the modules support that asynchronous simulation can integrate effectively into busy clinical training schedules without adding logistical strain. Faculty also independently sought access to the curriculum and reported similarly positive feedback, suggesting it may have utility beyond trainee education.

Although developed within TID, the underlying educational framework may be transferable to other clinical settings characterized by incomplete information, time pressure, and high‐stakes decisions. Within general ID fellowship training, this could be applied to common overnight pages, complex management decisions, blood‐borne pathogen exposures, and infection prevention questions. More broadly speaking, this model could be translated to other similar acute settings characterized by high‐stakes decisions such as emergency medicine and critical care, though this warrants further study. These findings suggest that asynchronous simulation may offer a practical approach for teaching complex clinical reasoning skills in settings where clinical exposure is inconsistent.

This study has several limitations. First, the curriculum was intentionally designed as a formative educational intervention. Outcomes were limited to Kirkpatrick Level 1 data, which reflect learner perceptions rather than objective measures of performance. Second, the sequential, gated design limited the ability to capture decision‐making or apply a scoring rubric. Restricting survey access to only those learners who completed all 12 modules may have introduced completion bias, and it is possible that participants were a self‐selected group of more motivated trainees with a greater baseline interest in TID and donor call evaluation. The number of trainees who received the invitation is unknown, and a response rate could not be calculated. While the platform tracks total sign‐ups we were unable to determine trainee dropout rates from outside faculty or duplicate accounts. In addition, because faculty self‐identified on the survey, data regarding years in practice were not obtained, which may have impacted how faculty perceived the curriculum relative to their own donor‐call experience. Third, donor call decision‐making is characterized by practice heterogeneity across institutions. Although cases were reviewed to ensure that acceptable responses reflected reasonable clinical approaches, differences in local practice may have influenced learner interpretation and applicability. Finally, given the small size of the dedicated TID subgroup, individual response variability likely had a noticeable impact on subgroup percentages, and these findings should be interpreted with caution. At the same time, however, the limited prior donor‐call exposure across the entire cohort suggests that this educational gap exists beyond dedicated TID fellowship training and may also affect general ID rotators on a TID service.

In conclusion, this multicenter pilot study demonstrates that an asynchronous, case‐based donor call simulation curriculum is a feasible approach to teaching clinical reasoning in TID. While this study primarily established feasibility and reach, future iterations of the curriculum should incorporate summative assessments within the platform to assess knowledge acquisition (Kirkpatrick Level 2) and explore translation to clinical practice (Kirkpatrick Level 3), including faculty‐observed donor call participation or performance in standardized simulation‐based assessments. The curriculum will continue to expand, with additional cases planned, and will remain freely accessible to ID and TID trainees, offering a scalable, low‐resource educational model with potential for broader application across transplant and other high‐stakes clinicaldomains.

## Author Contributions

**Robert Sean Tyler**: methodology, investigation, project administration, data curation, formal analysis, visualization, validation, writing – original draft, writing – review and editing. **Alexandra G. Mills**: validation, writing – review and editing**. Drew W. Charles**: validation, writing – review and editing. **Ruth O. Adekunle**: validation, writing – review and editing. **Divisha Sharma**: validation, writing – review and editing. **Yosra Alkabab**: validation, writing – review and editing. **Rachel B. Colven**: validation, writing – review and editing. **Scott R. Curry**: validation, writing – review and editing. **Ricardo M. La Hoz**: validation, writing – review and editing. **Courtney E. Harris**: conceptualization, methodology, investigation, project administration, data curation, formal analysis, supervision, visualization, validation, writing – original draft, writing – review and editing.

## Funding

The authors have nothing to report.

## Conflicts of Interest

The authors declare no conflicts of interest.

## Supporting information




**Supporting Information**: tid70284‐sup‐0001‐VisualAbstract.png
